# Supplementary material for: Chromothripsis during telomere crisis is independent of NHEJ, and consistent with a replicative origin
Source: Genome Res. 2019 May;29(5):737–49. doi: 10.1101/gr.240705.118 (PMC6499312; doi:10.1101/gr.240705.118)
Supplement: Supplemental Material [file supp_gr.240705.118_Supplemental_file_1.zip › contigs/annotated_contigs/DB57/contig.2.DB57_length_901_mean_cov_6.1653718091.docx]

**DB57_length_901_mean_cov_6.1653718091**

```
ATACTTGTCATATGTGATTCATTTTGGTGCTTCCCTCTTTCAGCACCTCTAAATAACGAATATCAAAGAAATAGGCTCAAAGAAATACA
 >chr7:145868277-145868509 - E=1e-127 p=4e-02
ATTAAATTTGCAAAAACTACCAAACGCAATAATTACCTAACTATACTATAAAATATTGTTTTGTCTCAAATATATTTTTTTCTACTTAG

TTCTTGTTTGCCTAGAGAGGTTATTTAAAGCTAAGAAGGATTGGTGATTGTCCA|A|TCCTGAAGTACTAAAGCCTGCTTAGTTTGGTT
                                                        >chr7:145870019-145870653 + E=0
TTCTGCAGCTGTGTGTGTGTTTTTTTTCTAATAAAATTGTTTTGTTTCGTTTTAATCCCTTAAAATTCCTATTAACATCAGGAAATATT
e+00
GATATAATCCTATCTGCTTTAAAAAGATTCTAAATGTTCAAATCTCCTCACCAGAACAAGTAGAAGTAAATCTGCTGAAAGGATTTTTA

AAAAAAACTGGCATTTTTAGTGAATTACGTGCAGAGAACTTTATATTTAGTTTTAATTTTGTTCAGTTACCTTAAGCCTTTTTTATCTT

ATTATACATTATTAGAGTAAATTTACATTTTTATCTAATCTCATATTAAGGATTGAATAATACACTTTTTGAAGTTTACAATAGTGTAC

ATATTATATGTATCTTGTTTTAATTTTCTCCAGGTGTTTTTAATAATTGAATTTTCCAGTTTGTTTGCTGTAAACATTGACCATTTAGA

AAATAAATTGATGTAACACTATCTGCTTCCTCAAATAATCCCCCTCACCTCCCCCCACACCACACACACTTATATCTTGCTTGTGTAGT

TTATAGCATAGTAAATATACATGCGTATTTTAGGGTATAAGGCTAAATTCAAATCTGTGATGCTATAT|TCTATATTTTTCCTGTTCTA

TTTTACATATTTAA
```
